# Effects of tucidinostat in adult T-cell leukemia/lymphoma in clinical practice

**DOI:** 10.1007/s12185-025-03963-9

**Published:** 2025-03-11

**Authors:** Ayako Kamiunten, Takuro Kameda, Masaaki Sekine, Hiroshi Kawano, Takanori Toyama, Keiichi Akizuki, Noriaki Kawano, Kouichi Maeda, Seiichi Sato, Masanori Takeuchi, Junzo Ishizaki, Koshiro Nagamine, Ayuka Kuroki, Ryoma Ikeda, Kengo Matsumoto, Masayoshi Karasawa, Yuki Tahira, Taisuke Uchida, Haruko Shimoda, Tomonori Hidaka, Kiyoshi Yamashita, Hideki Yamaguchi, Yoko Kubuki, Kazuya Shimoda, Kotaro Shide

**Affiliations:** 1 Division of Hematology, Diabetes, and Endocrinology, Department of Internal Medicine, Faculty of Medicine, University of Miyazaki, 5200 Kihara, Kiyotake, Miyazaki, 889-1692 Japan; 2 Koga General Hospital, 1749-1 Sudaki, Ikeuchi Machi, Miyazaki, 880-0041 Japan; 3 Miyazaki Prefectural Nobeoka Hospital, 2-1-10 Shinkouji, Nobeoka, 882-0835 Japan; 4 Miyazaki Prefectural Miyazaki Hospital, 5-30 Kitatakamatsu, Miyazaki, 880-8510 Japan; 5 Miyakonojo Medical Center, 5033-1 Iwayoshi-Cho, Miyakonojo, 880-8510 Japan; 6 Miyazaki Aisenkai Nichinan Hospital, 3649-2 Kazeta, Nichinan, 887-0034 Japan

**Keywords:** Adult T-cell leukemia/lymphoma, HDAC inhibitor, Tucidinostat

## Abstract

Adult T-cell leukemia/lymphoma (ATL) is a peripheral T-cell malignancy with a poor prognosis. We conducted a retrospective study across six institutions in Miyazaki Prefecture, Japan, to assess the efficacy of tucidinostat in patients with relapsed/refractory ATL who had not undergone transplantation. Between October 2021 and July 2023, 24 patients aged 41 to 88 years (median, 73.4 years) who had undergone prior therapies, including intensive chemotherapy (79.2%) and mogamulizumab immunotherapy (79.2%), received tucidinostat. Objective response rate (ORR), disease control rate (DCR), progression-free survival (PFS), and overall survival (OS) were evaluated as key outcomes. ORR and DCR reached 54.2% and 91.7%, respectively. The median PFS was 3.95 months, and OS was 8.04 months, which were not inferior to the results of a phase IIb study. The influential factors for PFS were age ≥ 75 years and high soluble IL-2 receptor (sIL-2R) levels above 5000 U/mL at the start of treatment. Favorable patients without these factors achieved a PFS of 11.4 months. Treatment-related adverse events were mainly hematologic but were managed over the course of treatment. Our findings indicate that tucidinostat provides survival benefits in patients with relapsed/refractory ATL in clinical practice and highlight key clinical factors for better outcomes.

**Supplementary Information:**

The online version contains supplementary material available at 10.1007/s12185-025-03963-9.

## Introduction

Adult T-cell leukemia/lymphoma (ATL) is an aggressive peripheral T-cell neoplasm characterized by the clonal proliferation of human T-cell leukemia virus type 1 (HTLV-1)-infected T cells [1]. ATL is classified into four subtypes, namely the acute, lymphoma, chronic, and smoldering types [2]. Acute, lymphoma, and chronic types with one or more unfavorable prognostic factors (high lactate dehydrogenase, high blood urea nitrogen, and low albumin) are defined as aggressive ATL [3]. The current standard therapy for aggressive ATLs is combination chemotherapy [4, 5]; specifically in patients aged < 70 years with aggressive ATL, the following VCAP-AMP-VECP regimen is the treatment standard: VCAP (vincristine (VCR), cyclophosphamide (CPA), doxorubicin (DXR), and prednisone (PSL)); AMP (DXR, ranimustine, and PSL); and VECP (vindesine, etoposide, carboplatin, and PSL) [4]. In addition, CPA, DXR, VCR, and PSL (CHOP)-like regimens are more frequently used in daily practice [6]. Katsuya et al. showed that the median overall survival (OS) was 8.3 months for acute-type ATL and 10.6 months for lymphoma-type ATL [7]. Our previous study reported that the median OS was 9 months for aggressive ATL when treated with combination chemotherapy [5].

The prognosis of ATL patients who are resistant to or relapse after combination chemotherapy (namely, relapsed/refractory ATL patients) is dismal. Mogamulizumab (a defucosylated anti-CC chemokine receptor 4 antibody), lenalidomide, tucidinostat, valemetostat, and brentuximab vedotin (if CD30 is expressed) can be prescribed for treating relapsed/refractory ATL patients in Japan [8]. Tucidinostat is a benzamide histone deacetylase (HDAC) inhibitor (HDACi) that selectively inhibits HDAC isoenzymes 1, 2, 3, and 10. Tucidinostat has been shown to induce apoptosis and inhibit the growth of ATL cell lines and primary tumor samples from ATL patients in vitro [9, 10]. A phase IIb clinical study of tucidinostat in relapsed/refractory ATL patients showed an ORR of 30.4% with a median PFS of 1.7 months [11]. Here, we report the effects of tucidinostat in patients with relapsed/refractory ATL in clinical practice.

## Methods

### Study design and participants

This retrospective study was conducted at the following six medical institutions in Miyazaki Prefecture, Japan: the University of Miyazaki Hospital, Koga General Hospital, Miyazaki Prefectural Nobeoka Hospital, Miyazaki Prefectural Miyazaki Hospital, Miyakonojo Medical Center Hospital, and Miyazaki Aisenkai Nichinan Hospital. This study aimed to evaluate the effect of tucidinostat on the survival outcomes of patients with relapsed, recurrent, or refractory ATL. Eligibility criteria were a confirmed diagnosis of ATL according to Shimoyama’s classification and administration of at least one dose of tucidinostat between October 2021 and July 2023. Relapsed ATL patients were defined as those who achieved complete remission (CR) or unconfirmed complete remission (CRu) with prior therapy but subsequently developed progressive disease (PD) [11]. Patients with recurrent ATL were defined as those who achieved partial remission (PR) or stable disease (SD) with prior therapy but subsequently developed PD [11]. Patients with refractory ATL were defined as those who did not respond to prior therapy, showed a reduction in tumor burden, or showed an improvement in symptoms [11]. Patients who had undergone hematopoietic stem cell transplantation before the study were excluded. In a previous phase IIb study, tucidinostat was initially administered at a dose of 40 mg twice weekly (40 mg BIW) and continued until disease progression or unacceptable toxicity [11]. In this retrospective study, initial dosing, dose adjustments, and treatment interruptions for all patients were managed according to the treating physician’s clinical practice. This study was approved by the Research Ethics Committee of the Faculty of Medicine, University of Miyazaki, and the ethics boards of all participating institutions.

### Data collection

Data were collected through a comprehensive review of medical records to identify patient characteristics, ATL subtypes, detailed treatment histories, responses to tucidinostat and prior therapies, adverse events (AEs), and survival outcomes.

### Outcome measures

The primary endpoints were PFS and OS. PFS was defined as the duration from the initiation of tucidinostat treatment to the first observed disease progression or death from any cause. OS is defined as the time from the start of tucidinostat treatment to death from any cause. Duration of response (DOR) was defined as the duration from the initial documented response (CR or PR) to disease progression or death from any cause. Secondary endpoints included ORR, disease control rate (DCR), and safety and tolerability of tucidinostat therapy. ORR was calculated as the proportion of patients who achieved either CR or PR. DCR was calculated as the proportion of patients achieving CR, PR, or SD. The efficacy of tucidinostat was further evaluated by assessing tumor response (CR or PR) at specific lesion sites, including nodal/extranodal lesions, skin lesions, and peripheral blood (PB) lesions (abnormal lymphocytes). Safety and tolerability were assessed by recording and analyzing AEs, which were categorized according to severity using the National Cancer Institute Common Terminology Criteria for Adverse Events version 5.0[12]. The highest toxicity grade was recorded for each patient during the treatment course.

### Statistical analysis

Descriptive statistics were used to summarize patient characteristics and provide an overview of the dataset. Some clinical factors were summarized with reference to cutoff values that have been previously reported for aggressive ATL [13, 14]. When comparisons between patient groups were necessary, categorical variables were evaluated using the Chi-square test or Fisher’s exact test, and continuous variables were evaluated using the *t* test or Mann–Whitney U test. Changes in tucidinostat dose, interruptions of treatment, changes in salvage therapy, and events during treatment such as response, AEs, or death were visualized using a swimmer plot. For survival analysis, PFS, OS, and DOR were evaluated using the Kaplan–Meier method, and differences in survival curves between subgroups were compared using the log-rank test. Cox proportional hazards models were used to examine the effects of covariates on survival outcomes following tucidinostat therapy. Adverse prognostic factors were weighted and stratified to predict survival. Statistical significance was set at a *p* value of less than 0.05. All statistical analyses and visualizations were performed using R software, version 4.4.

## Results

### Patient data

This study included 24 patients who were treated with at least one dose of tucidinostat over 2 years at six institutions in Miyazaki Prefecture, Japan. Patient characteristics are shown in Table 1. The median age at ATL diagnosis was 72 years (range, 41–85). The median age of patients at the start of tucidinostat treatment was 73.4 years (range, 41.6–88.0), and 33% of patients were aged more than 75 years. Patients had the following ATL subtypes: acute (n = 14, 58.3%), lymphoma (n = 6, 25.0%), and chronic with unfavorite factors (n = 4, 16.7%). The median time from initial ATL diagnosis to the start of tucidinostat treatment was 1.23 years (range, 0.18–5.04). The median time from the last treatment to the start of tucidinostat treatment was 30 days (range, 17–837). At the start of the tucidinostat treatment, six patients (25.0%) had Eastern Cooperative Oncology Group (ECOG) performance status (PS) ≥ 2. Seven patients (29.2%) had serum albumin levels less than 3.5 g/dL. Thirteen patients (54.2%) had sIL-2R levels greater than 5000 U/mL. Nodal or extranodal lesions, skin lesions, and PB lesions were observed in 15 (62.5%), 13 (54.2%), and 9 (37.5%) patients, respectively. Twenty-three patients (95.8%) had advanced stage with 3 or more. None of the patients had hypercalcemia greater than 11.0 mg/dL. All patients had received chemotherapy prior to tucidinostat therapy, with a median of two regimens (range, 1–5), and seven patients (29.2%) had a history of prior systemic therapy with three or more regimens. Nineteen patients (79.2%) had previously been treated with intensive chemotherapy. Nineteen patients (79.2%) had previously been treated with mogamulizumab. Seventeen patients (70.8%) were recurrent/refractory to their most recent therapies, and seven (29.2%) patients had relapsed disease (Table 1). Nineteen (79.2%) patients started treatment at the standard dose of 40 mg BIW, while five (21.8%) patients started with a reduced dose of 30 mg or lower BIW.

**Table 1:** Patient characteristics

Response to prior treatment				
Variable	Entire cohort	Relapse	Recurrent/refractory	p
Patients, N (%)	24	7 (29.2)	17 (70.8)	
Age at ATL Dx (M [R])	72.0 [41.0, 85.0]	73.0 [63.0, 84.0]	72.0 [41.0, 85.0]	0.525
Age at Tuc start (M [R])	73.4 [41.6, 88.0]	74.0 [65.6, 86.7]	73.3 [41.6, 88.0]	0.465
Age at Tuc start, ≥ 75, N (%)	8 (33.3)	3 (42.9)	5 (29.4)	0.647
Sex, female/male, N (%)	12/12 (50.0/50.0)	6/1 (85.7/14.3)	6/11 (35.3/64.7)	0.069
Subtype, N (%)				
Acute	14 (58.3)	3 (42.9)	11 (64.7)	
Lymphoma	6 (25.0)	2 (28.6)	4 (23.5)	
Unfavorable chronic	4 (16.7)	2 (28.6)	2 (11.8)	
Duration from ATL Dx to Tuc (y, M [R])	1.23 [0.18, 5.04]	1.49 [0.81, 3.00]	1.20 [0.18, 5.04]	0.634
Duration from prior Tx to Tuc (d, M [R])	30 [17, 837]	121 [17, 202]	31.5 [18, 837]	0.112
ECOG PS at Tuc start, 2–4, N (%)	6 (25.0)	1 (14.3)	5 (29.4)	0.629
Alb at Tuc start (g/dL), < 3.5, N (%)	7 (29.2)	2 (28.6)	5 (29.4)	1
sIL2R at Tuc start (U/mL), ≥ 5000, N (%)	13 (54.2)	2 (28.6)	11 (64.7)	0.182
Lesion site at Tuc start, N (%)				
Nodal/extranodal	15 (62.5)	2 (28.6)	13 (76.5)	0.061
Skin	13 (54.2)	4 (57.1)	9 (52.9)	1
Peripheral blood	9 (37.5)	3 (42.9)	6 (35.3)	1
Stage at Tuc start, 3–4, N (%)	23 (95.8)	7 (100)	16 (94.1)	1
cCa at Tuc start, mg/dL, M [R]	9.30 [7.40, 10.3]	9.25 [8.90, 9.90]	9.30 [7.40, 10.3]	0.785
Other blood test at Tuc start				
Neutrophil < 1500/μL, N (%)	4 (16.7)	2 (28.6)	2 (11.8)	0.552
Hemoglobin < 10 g/dL, N (%)	7 (29.2)	3 (42.9)	4 (23.5)	0.374
Platelet < 10 × 10^4^/μL, N (%)	2 (8.3)	0 (0.0)	2 (11.8)	1
T-Bil, mg/dL, M [R]	0.56 [0.23, 1.31]	0.57 [0.37, 0.75]	0.52 [0.23, 1.31]	1
ALP, U/L, M [R]	101 [40, 291]	91 [67, 131]	102 [40, 291]	0.461
Cre, mg/dL, M [R]	0.82 [0.49, 7.01]	0.89 [0.63, 1.06]	0.77 [0.49, 7.01]	0.703
Number of prior Txs, M [R]	2 [1–5]	2 [1–4]	2 [1–5]	
Number of prior Txs, N (%)				
1 therapy	8 (33.3)	2 (28.6)	6 (35.3)	1
2 therapies	9 (37.5)	3 (42.9)	6 (35.3)	
3 therapies	4 (16.7)	1 (14.3)	3 (17.6)	
4 therapies	2 (8.3)	1 (14.3)	1 (5.9)	
5 therapies	1 (4.2)	0 (0.0)	1 (5.9)	
Intensive CTx history, yes, N (%)	19 (79.2)	6 (85.7)	13 (76.5)	1
Mogamulizumab Tx history, yes, N (%)	19 (79.2)	5 (71.4)	14 (82.4)	0.608
Response to prior treatment, N (%)				
Relapse	7 (29.2)	7 (100)	0 (0.0)	< 0.001
Recurrent	8 (33.3)	0 (0.0)	8 (47.1)	
Refractory	9 (37.5)	0 (0.0)	9 (52.9)	
Initial dose of Tuc, N (%)				
10 mg BIW	1 (4.2)	0 (0.0)	1 (5.9)	1
20 mg BIW	3 (12.5)	1 (14.3)	2 (11.8)	
30 mg BIW	1 (4.2)	0 (0.0)	1 (5.9)	
40 mg BIW	19 (79.2)	6 (85.7)	13 (76.5)	

*N* number, *Tuc* tucidinostat, *y* year, *d* day, *M* median, *R* range, *Dx* diagnosis, *Tx* therapy, *ECOG PS* Eastern Cooperative Oncology Group performance status, *Alb* albumin, *sIL-2R* soluble interleukin-2 receptor, *cCa* corrected calcium, *BIW* twice weekly

### Efficacy

First, we examined ORR in the entire cohort and evaluated various clinical factors (Fig. S1). In the entire cohort, the ORR was 54.2% (95% CI: 34.2–74.1). Responses were observed across all ATL subtypes, with an ORR of 50.0% for acute-type ATL, 50.0% for lymphoma-type ATL, and 75.0% for unfavorable chronic-type ATL. Patients with sIL-2R levels < 5000 U/mL had significantly higher ORRs than those with higher levels. Patients who relapsed had a higher ORR than those who did not (recurrent or refractory), and patients younger than 75 years had a higher ORR than older patients, although these differences were not statistically significant. There were no differences in ORR based on ECOG PS (< 2 or ≥ 2), serum albumin level (< 3.5 or ≥ 3.5), stage (< 3 or ≥ 3), number of prior therapies (< 3 or ≥ 3), history of intensive chemotherapy, or history of mogamulizumab therapy (Fig. S1). The ORR varied depending on the site of the target lesion (Table 2). Specifically, the ORR was 33.3% (95% CI: 9.5–57.2) for nodal/extranodal lesions, 84.6% (95% CI: 65.0–100) for skin lesions, and 44.4% (95% CI: 12.0–76.9) for PB lesions.

**Table 2:** Response to tucidinostat treatment by target lesion type

ORR (%) (n/N, 95% CI)	Response of target lesions % (n/N, 95% CI)		
	Nodal/extranodal	Skin	Peripheral blood
54.2% (13/24, 34.2–74.1)	33.3% (5/15, 9.5–57.2)	84.6% (11/13, 65.0–100)	44.4% (4/9, 12.0–76.9)

*CI* confidence interval, *ORR* overall response rate

**Fig. 1:**
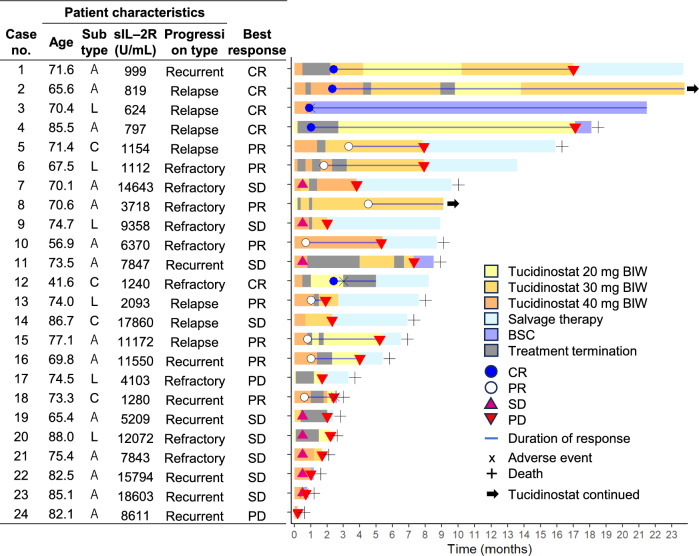
Treatment details and clinical courses of patients who received tucidinostat therapy. Swimmer plots showing treatment doses, treatment discontinuation, salvage therapy, and clinical outcomes. Horizontal bars indicate different doses of tucidinostat: 20, 30, and 40 mg BIW (twice weekly). Blue bars indicate salvage therapy. Circles and triangles represent patient responses, including complete response (CR), partial response (PR), stable disease (SD), and progressive disease (PD). Blue lines indicate the duration of response. X indicates an adverse event. + indicates patient death. Arrows indicate the continuation of tucidinostat therapy. The table on the left shows the clinical information at the start of tucidinostat treatment and the best response achieved for each patient

Next, we investigated the treatment details and clinical outcomes (Fig. 1). For all 24 patients, the median number of treatment cycles was two (range, 1–20), and the median duration of tucidinostat treatment was 74 days (range, 8–600 days). Seven patients (29.2%) received tucidinostat for more than 6 months. Twenty-three (95.8%) patients received treatment interruption or dose reduction, and the median time to the first interruption or dose reduction was 12 days (range, 7–42) or 47 days (range, 13–139), respectively (Fig. 1). Five (20.8%) achieved CR, and eight (33.3%) achieved PR. The disease control rate (DCR), which included CR, PR, and SD, was 91.7% (95% CI: 80.6–100). For the 13 patients who achieved an objective response (CR or PR), the median time to response was 1.0 month (95% CI: 1.10–2.40). Of these, eight patients (61.5%) required treatment interruption, and three patients (23%) required dose reduction prior to achieving an objective response. All nine patients who achieved SD required treatment interruption or dose reduction. The median dose for these responding patients (CR, PR, and SD) at the time of achieving these responses was 30 mg (range, 20–40).

**Fig. 2:**
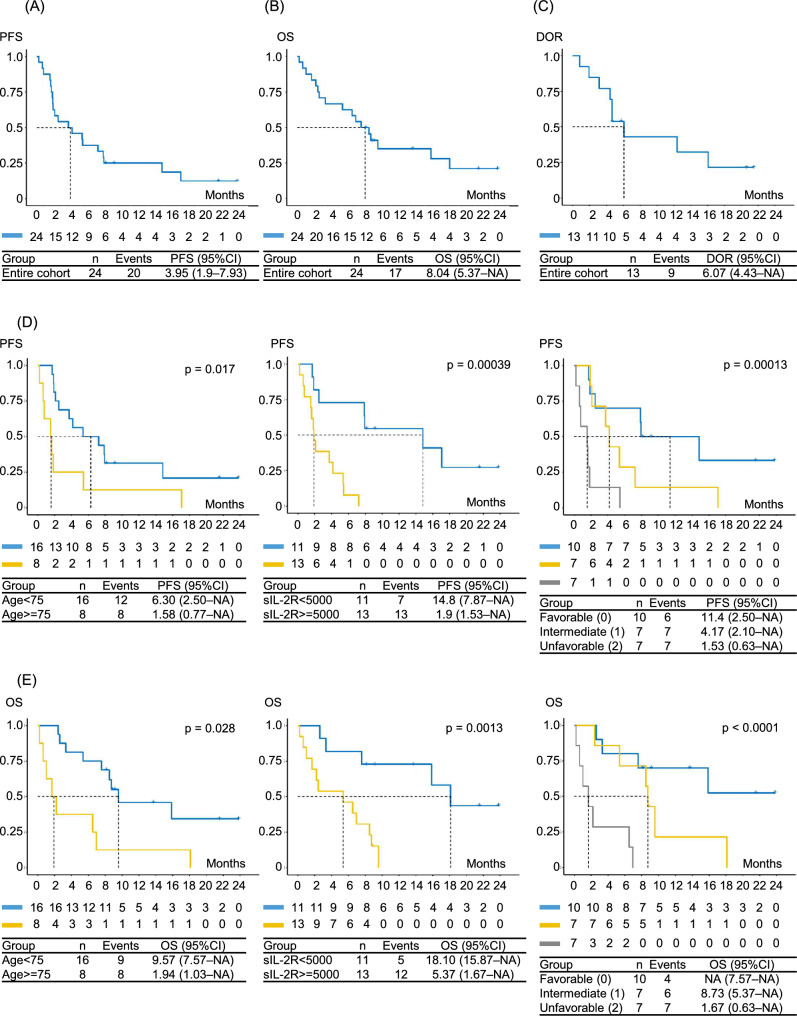
Survival outcomes following tucidinostat therapy. **A** Progression-free survival (PFS) after initiation of tucidinostat therapy determined using the Kaplan–Meier method. Number at risk and median survival time (MST) with 95% CI are shown. **B** Overall survival (OS) from the start of tucidinostat treatment to death or last follow-up. **C** Duration of response (DOR), defined as the time from the initial documented response (CR or PR) to disease progression or death from any cause, among patients who achieved CR or PR. **D** PFS stratified by a prognostic model based on the total number of adverse factors (older age (≥ 75 years) and higher sIL-2R level (≥ 5000 U/mL) at the start of tucidinostat. **E** OS stratified according to this prognostic model

The median PFS was 3.95 months (95% CI: 1.9–7.93) (Fig. 2A). Eighteen patients (75.0%) interrupted tucidinostat treatment because of disease progression (n = 14), COVID-19 pneumonia (n = 1, case 5), cryptococcal meningitis (n = 1, case 23), and AEs (skin rash) (n = 2, case 3 and 12), with a median follow-up duration of 1.09 years. Twelve patients received subsequent treatment with valemetostat (n = 5), gemcitabine, dexamethasone, and cisplatin (GDP) therapy (n = 5), etoposide (n = 3), or mogamulizumab (n = 3), and ten received the best supportive care only. Two patients with skin rashes maintained a tumor response without salvage therapies.

The median OS was 8.04 months (95% CI: 5.37–NA) (Fig. 2B). Four patients (16.7%) died during tucidinostat therapy, all of whom were classified as having PD at the time of death. Two (8.3%) continued tucidinostat therapy; one (case 2) achieved CR and the other (case 8) achieved PR.

For the 13 patients who achieved objective response (CR or PR), the DOR was 6.07 months (4.43–NA) (Fig. 2C).

### Prognostic factors for tucidinostat therapy

Given the relatively small number of patients (n = 24), univariate Cox regression analysis was performed on PFS using clinical factors at the start of tucidinostat therapy (age ≥ 75, ECOG PS ≥ 2, stage ≥ 3, albumin < 3.5 g/dL, each 5000 U/mL increase in sIL-2R level, recurrent/refractory disease type, number of prior therapies, history of intensive chemotherapy, and history of mogamulizumab therapy) (Table 3). Among these factors, older age and higher sIL-2R level (per 5000 U/mL increase) were found to adversely affect PFS. When patients were stratified based on the level of each factor, PFS and OS were well stratified by older age (≥ 75 years) and higher sIL-2R level (≥ 5000 U/mL) (Fig. 2D, E) but were not stratified by other factors (PS, stage, albumin, disease type, number of prior therapies, prior intensive chemotherapy, or prior mogamulizumab therapy) (Fig. S2A, B). Furthermore, a prognostic model based on the total number of the two adverse factors (age ≥ 75 years and sIL-2R level ≥ 5000 U/mL) demonstrated distinct prognostic groups (Fig. 2D, E). In the favorable group, PFS and OS were 11.4 (2.50–NA) and NA (7.57–NA), respectively.

**Table 3:** Univariate Cox regression analyses of PFS (n = 24)

Variable	Univariate	
	HR (95% CI)	p
Age ≥ 75 at tucidinostat start	2.87 (1.15–7.17)	0.024
ECOG PS ≥ 2 at tucidinostat start	1.45 (0.51–4.14)	0.484
Stage ≥ 3 at tucidinostat start	2.61 (0.60–11.3)	0.200
Alb < 3.5 g/dL at tucidinostat start	1.17 (0.45–3.06)	0.753
sIL-2R increase of 5000 U/mL	2.51 (1.49–4.23)	< 0.001
Recurrent/refractory disease	2.65 (0.86–8.21)	0.090
Number of prior therapies	1.1 (0.729–1.65)	0.657
History of ≥ 2 prior therapies	0.85 (0.334–2.17)	0.733
History of ≥ 3 prior therapies	1.73 (0.68–4.43)	0.254
History of ≥ 4 prior therapies	1.02 (0.293–3.52)	0.981
History of intensive chemotherapy	1.05 (0.35–3.16)	0.936
History of mogamulizumab therapy	0.64 (0.23–1.78)	0.391

*PFS* progression-free survival, *Alb* albumin, *sIL-2R* soluble interleukin-2 receptor, *ECOG PS* Eastern Cooperative Oncology Group performance status, *p p* value, *HR* hazard ratio, *CI* confidence interval

### Subgroup analysis

We performed a subgroup analysis of ORR, PFS, and OS in patients with recurrent/refractory disease at the start of tucidinostat therapy (n = 17). Baseline characteristics were similar between patients with recurrent/refractory disease and those with relapsed disease, with no significant differences observed (Table 1). The ORR of this subgroup was 41.2% (17.8–64.6%) (Fig. S3). Patients with sIL-2R levels < 5000 U/mL had significantly higher ORRs of 83.3% (53.5–100%) than those with higher levels. Patients younger than 75 years had higher ORRs of 58.3% (30.4–86.2%) than those who were not younger. In addition, PFS and OS reached 2.5 (1.7–7.87) and 5.37 (2.43–NA), respectively (Fig. S4A, B), and there was no significant difference from those in the entire cohort, including patients with relapse disease. Furthermore, PFS and OS were stratified in the same manner using the prognostic model developed for the entire cohort (Fig. S4C, D).

### Safety and tolerability

Tucidinostat-related AEs were observed in 24 patients (Table 4). The most common AEs (all grades) were decreased platelet and neutrophil count. Of these, 19 (79.1%) patients experienced grade ≥ 3 AEs, with 17 (70.8%) having hematological AEs (thrombocytopenia (n = 12, 50%), neutropenia (n = 11, 45.9%), anemia (n = 2, 8.3%)), and 5 (20.8%) having non-hematological AEs. Twenty-three (95.8%) patients required dose adjustment, of which 13 (54.2%) required dose reductions and 18 (75%) required dose interruptions. All hematologic AEs were manageable with dose adjustments and supportive care, including platelet transfusion in six patients and granulocyte colony stimulating factor in 11 patients. Treatment interruptions were required in 12 (50%) patients due to grade ≥ 3 thrombocytopenia and in 11 (45.9%) patients due to grade ≥ 3 neutropenia, though no permanent discontinuations were required. Regarding non-hematological AEs, two (8.3%) patients discontinued tucidinostat due to grade 3 and grade 2 rashes. In addition, two (8.3%) patients discontinued due to grade 4 cryptococcal meningitis and grade 2 pneumonia infections.

**Table 4:** Summary of AEs during tucidinostat therapy

AE type	Entire cohort (n = 24)			30 mg BIW (n = 5)			40 mg BIW (n = 19)		
	All grades, n (%)	Grade > = 3, n (%)	Dose reduction/treatment interruption, n (%)	All grades, n (%)	Grade > = 3, n (%)	Dose reduction/treatment interruption, n (%)	All grades, n (%)	Grade > = 3, n (%)	Dose reduction/treatment interruption, n (%)
Overall AEs	24 (100)	19 (79.1)	23 (95.8)	5 (100)	5 (100)	4 (80.0)	19 (100)	14 (73.7)	18 (94.7)
Hematological AEs	21 (87.5)	17 (70.8)	19 (79.1)	5 (100)	5 (100)	4 (80.0)	17 (89.4)	12 (63.2)	14 (73.7)
Platelet count decreased	18 (75.0)	12 (50.0)	12 (50.0)	4 (80.0)	3 (60.0)	3 (60.0)	14 (73.7)	9 (47.4)	9 (47.4)
Neutrophil count decreased	15 (62.5)	11 (45.9)	11 (45.9)	5 (100)	3 (60.0)	3 (60.0)	10 (52.6)	8 (42.2)	8 (42.2)
Anemia	10 (41.7)	2 (8.3)	2 (8.3)	2 (40.0)	1 (20.0)	1 (20.0)	8 (42.1)	1 (5.3)	1 (5.3)
Non-hematological AEs	18 (75.0)	5 (20.8)	4 (16.7)	3 (60.0)	0	0	13 (68.4)	5 (26.3)	4 (21.1)
Skin rash	2 (8.3)	1 (4.2)	2 (8.3)*	0	0		2 (10.5)	1 (5.3)	2 (10.5)*
Infections	3 (12.5)	1 (4.2)	2 (8.3)**	1 (20.0)	0		2 (10.5)	1 (5.3)	2 (10.5)**
Gastrointestinal disorders	9 (37.5)	2 (8.3)		1 (20.0)	0		8 (42.1)	2 (10.5)	
Nausea/vomiting	7 (29.2)	0		0	0		7 (36.8)	0	
Decreased appetite	7 (29.2)	0		3 (60.0)	0		4 (21.1)	0	
Mucositis oral	3 (12.5)	0		1 (20.0)	0		2 (10.5)	0	
Diarrhea	1 (4.2)	1 (4.2)		0	0		1 (5.3)	0	
Fatigue	2 (8.3)	0		1 (20.0)	0		1 (5.3)	0	
Dysgeusia	1 (4.2)	0		0	0		1 (5.3)	0	
Hepatobiliary disorders	4 (16.7)	1 (4.2)		0	0		4 (21.1)	1 (5.3)	

*AEs* adverse events, *BIW* twice weekly

^*^treatment discontinued due to grade 3 (case 3) and grade 2 rash (case 12)

^**^treatment discontinued due to grade 4 cryptococcal meningitis (case 23) and grade 2 pneumonia (case 18)

In patients receiving the standard starting dose of 40 mg BIW, as used in the Phase IIb study, the overall incidence of grade 3–4 AEs was 73.7% (n = 14) (Table 4). Specifically, grade 3–4 hematologic AEs occurred in 63.2% (n = 12), while grade 3–4 non-hematologic AEs occurred in 26.3% (n = 5). The discontinuation rate in this subgroup was 21.1% (n = 4), with all discontinuations occurring due to non-hematologic AEs (two rashes and two infections).

## Discussion

This retrospective analysis reports the therapeutic efficacy of tucidinostat for the treatment of relapsed/refractory ATL in clinical practice. All patients experienced disease progression after their most recent previous therapies, and the majority had received intensive multidrug chemotherapy and mogamulizumab immunotherapy prior to tucidinostat therapy. The ORR was 54.2% and 20.8% achieved CR, and median PFS and OS were 3.95 and 8.04 months, respectively. These values were not inferior to those reported in a phase IIb study planned for patients with relapsed/refractory ATL, in which the ORR and CR rates were 30% and 4.3%, and the median PFS and OS were 1.7 and 7.9 months, respectively [11].

Clinical trials are the most rigorous way to test how novel treatments compare with existing treatments for a given outcome. However, the results obtained from clinical trials are often not reproduced in clinical practice, mainly due to differences in patient therapy histories, PS, and organ function. For example, the ORR and CR rates achieved with mogamulizumab therapy in relapsed/refractory ATL patients in clinical practice were only 36% and 17%, whereas these rates were 50% and 31%, respectively, in a phase 2 study [15]. The median PFS and OS for relapsed/refractory ATL patients in clinical practice from the start of mogamulizumab therapy were 1.8 and 4.0 months, respectively, while those in the same clinical trial were 5.2 and 13.7 months, respectively. In the post-marketing surveillance program of mogamulizumab with a large number of patients, a shorter OS (5.5 months) from the start of mogamulizumab in patients with relapsed/refractory ATL compared with that in a phase IIb study was also reported [16].

Univariate Cox regression analysis showed that older age and higher sIL-2R levels at the start of tucidinostat negatively influenced PFS with tucidinostat. There was no major difference in the proportion of patients aged 75 years or older between our cohort and the phase 2 cohort, with 67% and 39%, respectively. Tucidinostat demonstrated non-inferiority for ORR, PFS, and OS regardless of the type of progression at the start of tucidinostat treatment; ORR, PFS, and OS in the recurrent/refractory subgroup in our cohort were not inferior to those seen in all patients enrolled in the phase IIb clinical trial. The higher ORR and longer PFS associated with tucidinostat therapy in the recurrent/refractory subgroup, along with the absence of poor effect on PFS by the number of previous therapy lines, indicate the therapeutic potential of tucidinostat in clinical practice. The therapeutic potential of tucidinostat independent of refractory status or previous treatment history has also been reported in other studies [11, 17].

It should also be noted that treatment options were not exhausted in patients who exhibited progression while undergoing tucidinostat treatment. Of the 20 patients who progressed to PD during tucidinostat therapy, 12 received other therapies, including valemetostat [18, 19]; some responded to these subsequent therapies. As the prognosis of relapsed/refractory ATL is dismal, with a median OS of 125 days and 1-year OS of 18% (which is especially low at 13% for patients who do not undergo allo-HSCT) [20], utilizing sequential therapies in clinical practice may have contributed to the improved 1-year OS of 36% after tucidinostat therapy reported herein.

The side effects associated with tucidinostat were manageable with medical intervention. Rates of grade >  = 3 AEs and treatment discontinuation in patients with 40 mg BIW in this study were similar to those observed in the Phase IIb study, supporting the manageable safety profile at this dose. However, as dose reduction or treatment interruption was required in 71% of patients due to AEs, close monitoring, effective supportive care, active management, and flexible dose adjustment may be necessary to ensure treatment continuation.

This study has some limitations, primarily due to the small sample size. In addition, the impact of subsequent treatments, which could affect prognosis after tucidinostat therapy, could not be assessed. Further clinical observations with larger numbers of patients would be warranted to clarify these points.

## Conclusion

Tucidinostat has shown promising efficacy in clinical practice and may prolong the PFS and OS of patients. Considering significant clinical factors (sIL-2R level and age) before beginning tucidinostat administration may aid in the identification of optimal patients for this treatment strategy.

## Supplementary Information

Below is the link to the electronic supplementary material.

Supplementary file1 (PDF 740 kb)

## Data Availability

Please contact Takuro Kameda (takuro_kameda@med.miyazaki-u.ac.jp) for requests for data sharing.
