# Supplementary material for: Effects of tucidinostat in adult T-cell leukemia/lymphoma in clinical practice
Source: Int J Hematol. 2025 Mar 11;122(1):83–92. doi: 10.1007/s12185-025-03963-9 (PMC12202688; doi:10.1007/s12185-025-03963-9)
Supplement: Supplementary file 1 — Supplementary file1 (PDF 740 kb) [file 12185_2025_3963_MOESM1_ESM.pdf]

Supplementary Material

Table S1. Patient characteristics stratified by initial dose of tucidinostat.

	Initial dose of tucidinostat (BIW)		
**Variable**	**30mg or lower**	**40mg**	**p**
Patients, N (%)	5 (20.8)	19 (79.2)	
Age at ATL Dx (M [R])	74.0 [64.0, 85.0]	72.0 [41.0, 85.0]	0.393
Age at Tuc start (M [R])	74.5 [65.4, 88.0]	73.3 [41.6, 86.7]	0.455
Age at Tuc start, ≥75, N (%)	2 (40.0)	6 (31.6)	1
Sex, female/male, N (%)	3/2 (60.0/40.0)	9/10 (47.4/52.6)	1
Subtype, N (%)			0.653
Acute	3 (60.0)	11 (57.9)	
Lymphoma	2 (40.0)	4 (21.1)	
Unfavorable chronic	0 (0.0)	4 (21.1)	
Duration from ATL Dx to Tuc (y, M [R])	0.93 [0.60, 2.67]	1.25 [0.18, 5.04]	0.943
Duration from prior Tx to Tuc (d, M [R])	17.5 [17.0, 29.0]	87.5 [23.0, 837.0]	0.019
ECOG PS at Tuc start, 2–4, N (%)	3 (60.0)	3 (15.8)	0.078
Alb at Tuc start (g/dL), < 3.5, N (%)	2 (40.0)	5 (26.3)	0.608
sIL2R at Tuc start (U/mL), ≥5000, N (%)	2 (40.0)	11 (57.9)	0.63
Lesion site at Tuc start, N (%)			
Nodal/extranodal	4 (80.0)	11 (57.9)	0.615
Skin	3 (60.0)	10 (52.6)	1
Peripheral blood	1 (20.0)	8 (42.1)	0.615
Stage at Tuc start, 3–4, N (%)	4 (80.0)	19 (100)	0.208
cCa at Tuc start, mg/dL, M [R]	9.57 [7.40, 10.0]	9.28 [8.76, 10.3]	0.385
Other blood test at Tuc start			
Neutrophil < 1500/μL, N (%)	2 (40.0)	2 (10.5)	0.179
Hemoglobin < 10 g/dL, N (%)	4 (80.0)	3 (15.8)	0.014
Platelet < 10×10^4/μL, N (%)	2 (40.0)	0 (0.0)	0.036
T-Bil, mg/dL, M [R]	0.60 [0.23, 0.90]	0.54 [0.27, 1.31]	0.772
ALP, U/L, M [R]	169 [108, 291]	91 [40, 142]	0.014
Cre, mg/dL, M [R]	1.04 [0.49, 7.01]	0.82 [0.56, 1.53]	0.696
Number of prior Txs, M [R]	2 [1–5]	2 [1–4]	
Number of prior Txs, N (%)			0.219
1 therapy	2 (40.0)	6 (31.6)	
2 therapies	1 (20.0)	8 (42.1)	
3 therapies	0 (0.0)	4 (21.1)	
4 therapies	1 (20.0)	1 (5.3)	
5 therapies	1 (20.0)	0 (0.0)	
Intensive CTx history, yes, N (%)	4 (80.0)	15 (78.9)	1
Mogamulizumab Tx history, yes, N (%)	3 (60.0)	16 (84.2)	0.27
Response to prior treatment, N (%)			0.567
Relapse	1 (20.0)	6 (31.6)	
Recurrent	1 (20.0)	7 (36.8)	
Refractory	3 (60.0)	6 (31.6)	

Abbreviations: N, number; Tuc, tucidinostat; y, year; d, day; M, median; R, range; Dx, diagnosis; Tx, therapy; ECOG PS, Eastern Cooperative Oncology Group performance status; Alb, albumin; sIL-2R, soluble interleukin-2 receptor; cCa, corrected calcium; BIW, twice weekly.

Supplemental Figures

Figure S1.

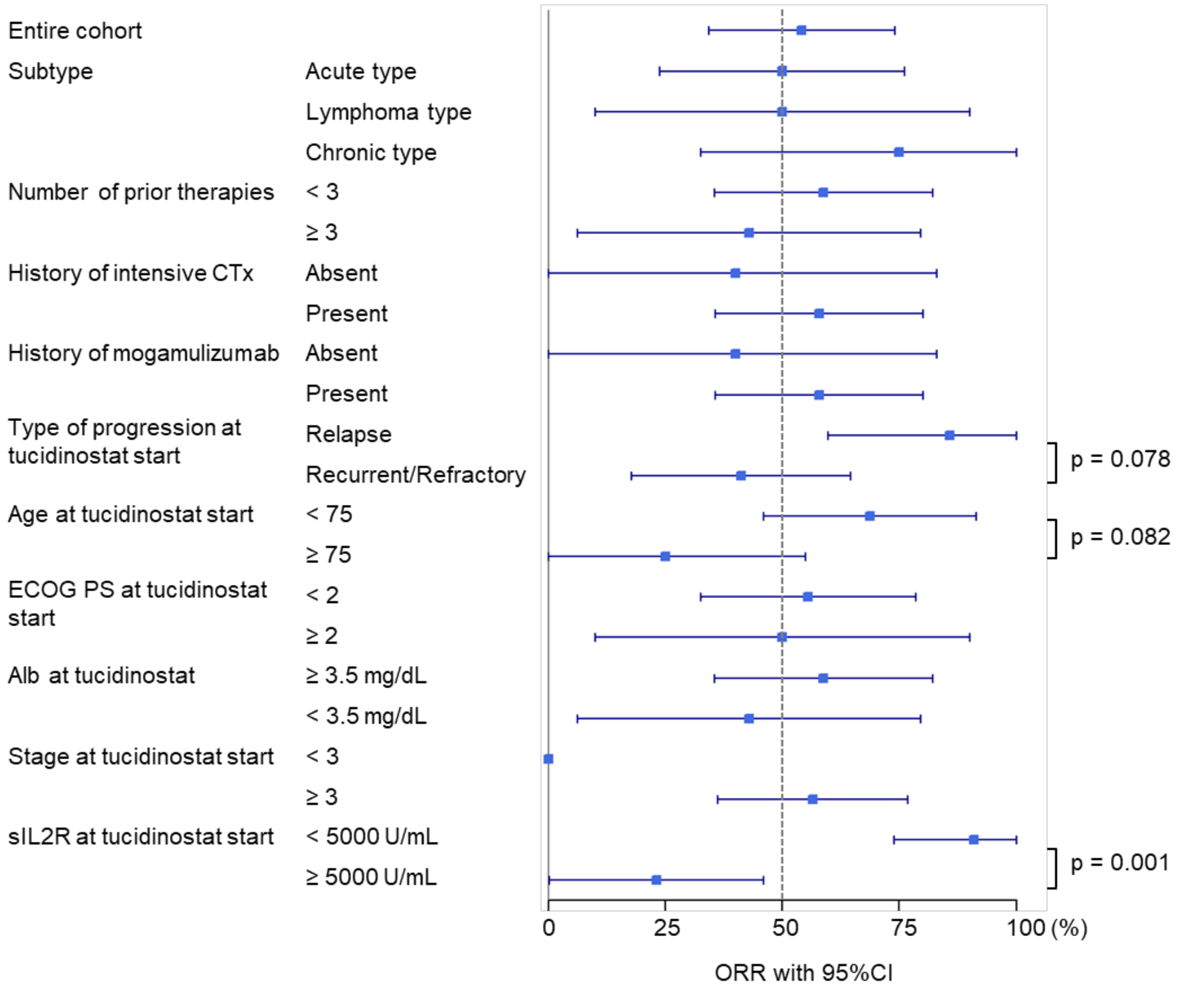

Figure S1. Objective response rate (ORR) by clinical variables following tucidinostat therapy.

Forest plot depicting the ORR of tucidinostat therapy using clinical variables. The blue squares represent the estimated ORR and the horizontal lines denote the 95% confidence intervals.

Figure S2. (A)

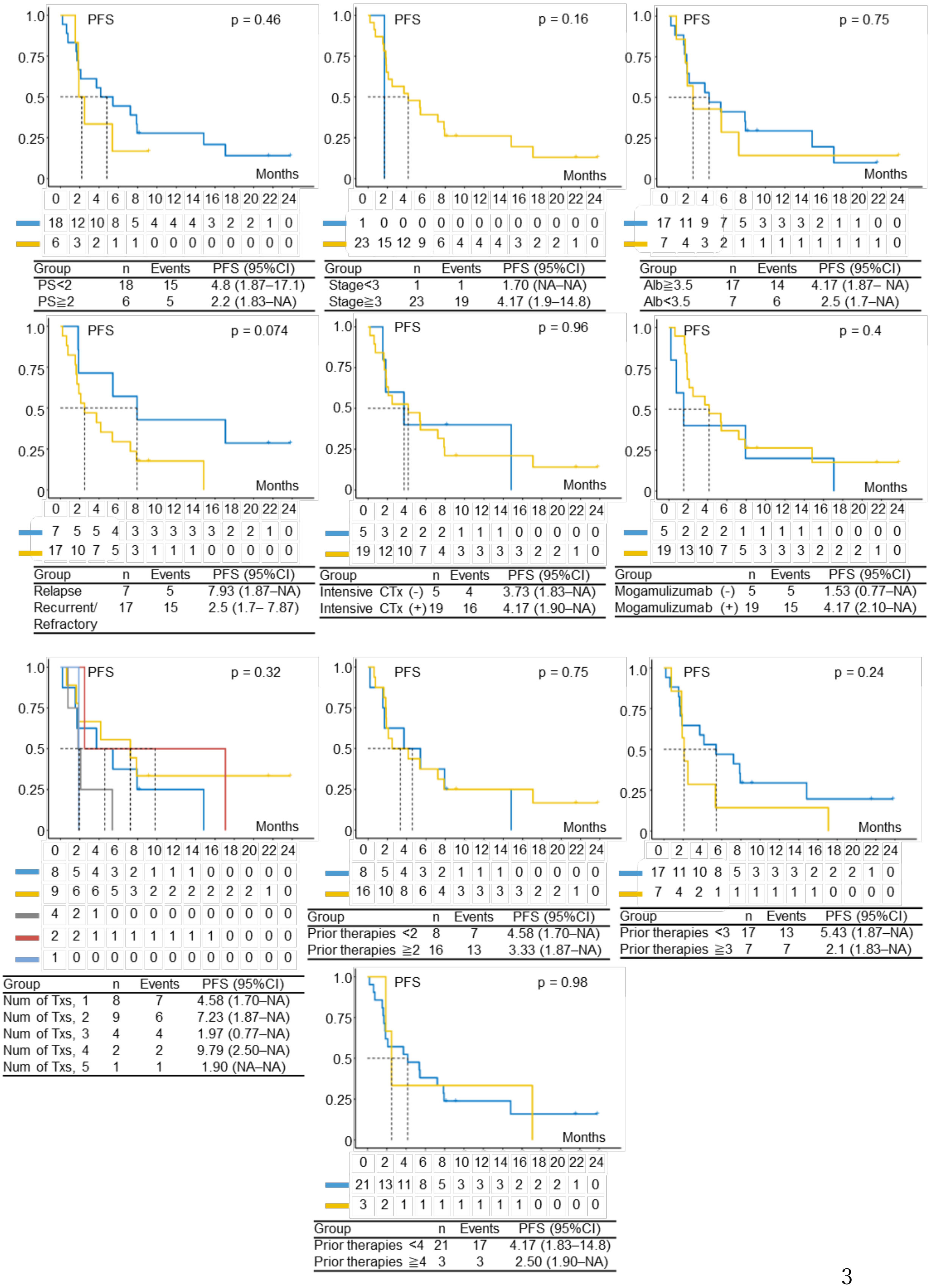
PFS p = 0.46
Months
0 2 4 6 8 10 12 14 16 18 20 22 24
18 12 10 8 5 4 4 4 3 2 2 1 0
6 3 2 1 1 0 0 0 0 0 0 0 0
Group n Events PFS (95%CI)
PS<2 18 15 4.8 (1.87–17.1)
PS≧2 6 5 2.2 (1.83–NA)
PFS p = 0.16
1 0 0 0 0 0 0 0 0 0 0 0 0
23 15 12 9 6 4 4 4 3 2 2 1 0
Group n Events PFS (95%CI)
Stage<3 1 1 1.70 (NA–NA)
Stage≧3 23 19 4.17 (1.9–14.8)
PFS p = 0.75
17 11 9 7 5 3 3 3 2 1 1 0 0
7 4 3 2 1 1 1 1 1 1 1 1 0
Group n Events PFS (95%CI)
Alb≧3.5 17 14 4.17 (1.87– NA)
Alb<3.5 7 6 2.5 (1.7–NA)
PFS p = 0.074
7 5 5 4 3 3 3 3 3 2 2 1 0
17 10 7 5 3 1 1 1 0 0 0 0 0
Group n Events PFS (95%CI)
Relapse 7 5 7.93 (1.87–NA)
Recurrent/ Refractory 17 15 2.5 (1.7– 7.87)
PFS p = 0.96
5 3 2 2 2 1 1 1 0 0 0 0 0
19 12 10 7 4 3 3 3 3 2 2 1 0
Group n Events PFS (95%CI)
Intensive CTx (-) 5 4 3.73 (1.83–NA)
Intensive CTx (+) 19 16 4.17 (1.90–NA)
PFS p = 0.4
5 2 2 2 1 1 1 1 1 0 0 0 0
19 13 10 7 5 3 3 3 2 2 2 1 0
Group n Events PFS (95%CI)
Mogamulizumab (-) 5 5 1.53 (0.77–NA)
Mogamulizumab (+) 19 15 4.17 (2.10–NA)
PFS p = 0.32
8 5 4 3 2 1 1 1 0 0 0 0 0
9 6 6 5 3 2 2 2 2 2 2 1 0
4 2 1 0 0 0 0 0 0 0 0 0 0
2 2 1 1 1 1 1 1 1 0 0 0 0
1 0 0 0 0 0 0 0 0 0 0 0 0
Group n Events PFS (95%CI)
Num of Txs, 1 8 7 4.58 (1.70–NA)
Num of Txs, 2 9 6 7.23 (1.87–NA)
Num of Txs, 3 4 4 1.97 (0.77–NA)
Num of Txs, 4 2 2 9.79 (2.50–NA)
Num of Txs, 5 1 1 1.90 (NA–NA)
PFS p = 0.75
8 5 4 3 2 1 1 1 0 0 0 0 0
16 10 8 6 4 3 3 3 3 2 2 1 0
Group n Events PFS (95%CI)
Prior therapies <2 8 7 4.58 (1.70–NA)
Prior therapies ≧2 16 13 3.33 (1.87–NA)
PFS p = 0.24
17 11 10 8 5 3 3 3 2 2 2 1 0
7 4 2 1 1 1 1 1 1 0 0 0 0
Group n Events PFS (95%CI)
Prior therapies <3 17 13 5.43 (1.87–NA)
Prior therapies ≧3 7 7 2.1 (1.83–NA)
PFS p = 0.98
21 13 11 8 5 3 3 3 2 2 2 1 0
3 2 1 1 1 1 1 1 1 0 0 0 0
Group n Events PFS (95%CI)
Prior therapies <4 21 17 4.17 (1.83–14.8)
Prior therapies ≧4 3 3 2.50 (1.90–NA)

Figure S2. (B)

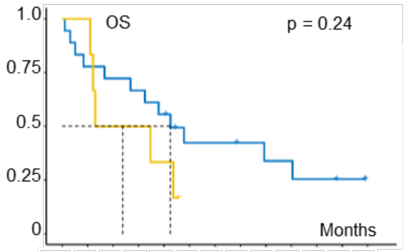

	0	2	4	6	8	10	12	14	16	18	20	22	24
	18	14	13	12	10	6	6	5	4	4	3	2	0
	6	6	3	3	2	0	0	0	0	0	0	0	0

Group	n	Events	OS (95%CI)
PS<2	18	12	8.5 (5.37–NA)
PS≧2	6	5	4.76 (2.43–NA)

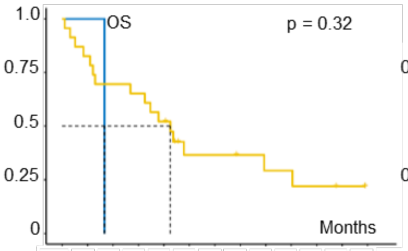

	0	2	4	6	8	10	12	14	16	18	20	22	24
	1	1	0	0	0	0	0	0	0	0	0	0	0
	23	19	16	15	12	6	6	5	4	4	3	2	0

Group	n	Events	OS (95%CI)
Stage<3	1	1	3.33 (NA–NA)
Stage≧3	23	16	8.50 (5.37–NA)

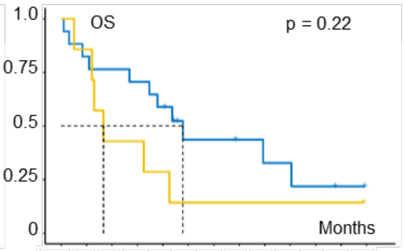

	0	2	4	6	8	10	12	14	16	18	20	22	24
	17	14	13	12	10	5	5	4	3	3	2	1	0
	7	6	3	3	2	1	1	1	1	1	1	1	0

Group	n	Events	OS (95%CI)
Alb≧3.5	17	11	9.57 (6.93–NA)
Alb<3.5	7	6	3.33 (2.43–NA)

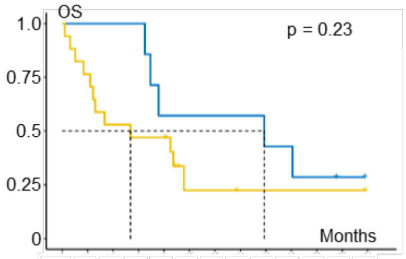

	0	2	4	6	8	10	12	14	16	18	20	22	24
	7	7	7	7	4	4	4	4	3	3	2	1	0
	17	13	9	8	8	2	2	1	1	1	1	1	0

Group	n	Events	OS (95%CI)
Relapse	7	5	15.87 (6.93–NA)
Recurrent/ Refractory	17	15	5.37 (2.43–NA)

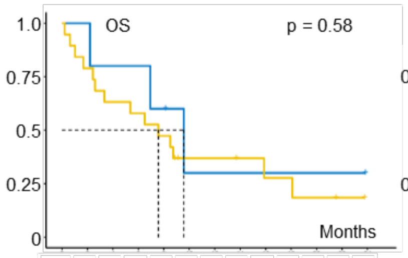

	0	2	4	6	8	10	12	14	16	18	20	22	24
	5	5	4	4	3	1	1	1	1	1	1	1	0
	19	15	12	11	9	5	5	4	3	3	2	1	0

Group	n	Events	OS (95%CI)
Intensive CTx (-)	5	3	9.57 (6.93–NA)
Intensive CTx (+)	19	14	7.57 (3.33–NA)

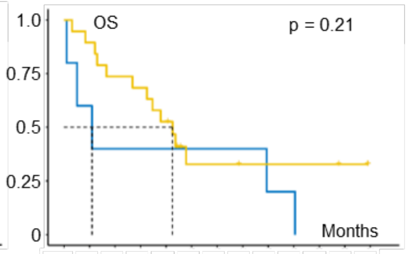

	0	2	4	6	8	10	12	14	16	18	20	22	24
	5	3	2	2	2	2	2	2	1	1	0	0	0
	19	17	14	13	10	4	4	3	3	3	3	2	0

Group	n	Events	OS (95%CI)
Mogamulizumab (-)	5	5	2.2 (1.03–NA)
Mogamulizumab (+)	19	12	8.5 (6.50–NA)

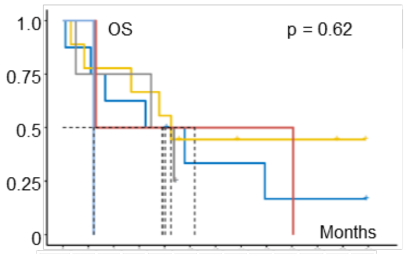

	0	2	4	6	8	10	12	14	16	18	20	22	24
	8	7	5	5	4	2	2	2	1	1	1	1	0
	9	7	7	6	5	3	3	2	2	2	2	1	0
	4	3	3	3	2	0	0	0	0	0	0	0	0
	2	2	1	1	1	1	1	1	1	1	0	0	0
	1	1	0	0	0	0	0	0	0	0	0	0	0

Group	n	Events	OS (95%CI)
Num of Txs, 1	8	6	8.04 (3.33–NA)
Num of Txs, 2	9	5	8.50 (5.37–NA)
Num of Txs, 3	4	3	7.83 (1.03–NA)
Num of Txs, 4	2	2	10.35 (2.60–NA)
Num of Txs, 5	1	1	2.43 (NA–NA)

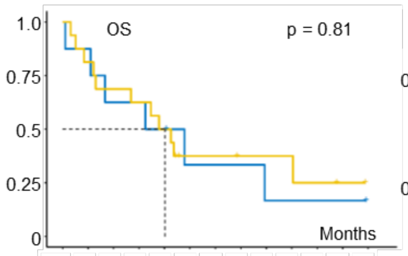

	0	2	4	6	8	10	12	14	16	18	20	22	24
	8	7	5	5	4	2	2	2	1	1	1	1	0
	16	13	11	10	8	4	4	3	3	3	2	1	0

Group	n	Events	OS (95%CI)
Prior therapies <2	8	6	8.04 (3.33–NA)
Prior therapies ≧2	16	11	8.04 (2.60–NA)

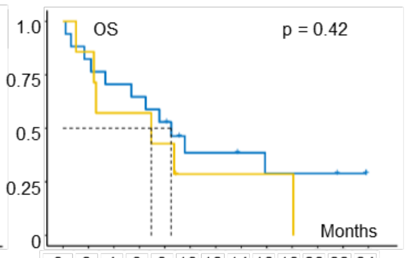

	0	2	4	6	8	10	12	14	16	18	20	22	24
	17	14	12	11	9	5	5	4	3	3	3	2	0
	7	6	4	4	3	1	1	1	1	1	0	0	0

Group	n	Events	OS (95%CI)
Prior therapies <3	17	11	8.50 (5.37–NA)
Prior therapies ≧3	7	6	6.93 (2.43–NA)

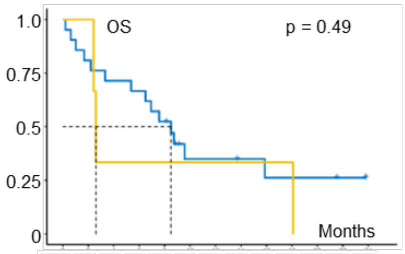

	0	2	4	6	8	10	12	14	16	18	20	22	24
	21	17	15	14	11	5	5	4	3	3	3	2	0
	3	3	1	1	1	1	1	1	1	1	0	0	0

Group	n	Events	OS (95%CI)
Prior therapies <4	21	14	8.5 (5.37–NA)
Prior therapies ≧4	3	3	2.6 (2.43–NA)

Figure S2. Survival outcomes stratified by clinical factors following tucidinostat therapy.

(A) Progression-free survival (PFS) after the initiation of tucidinostat therapy stratified by clinical factors, such as ECOG PS, stage, albumin, type of disease progression, number of prior therapies, history of intensive chemotherapy, and history of mogamulizumab therapy. The number of patients at risk and median survival time (MST) with 95% CI are shown. The log-rank test was used to compare the differences between groups. (B) Overall survival (OS) from the initiation of tucidinostat treatment to death or last follow-up stratified by the same clinical factors.

Figure S3.

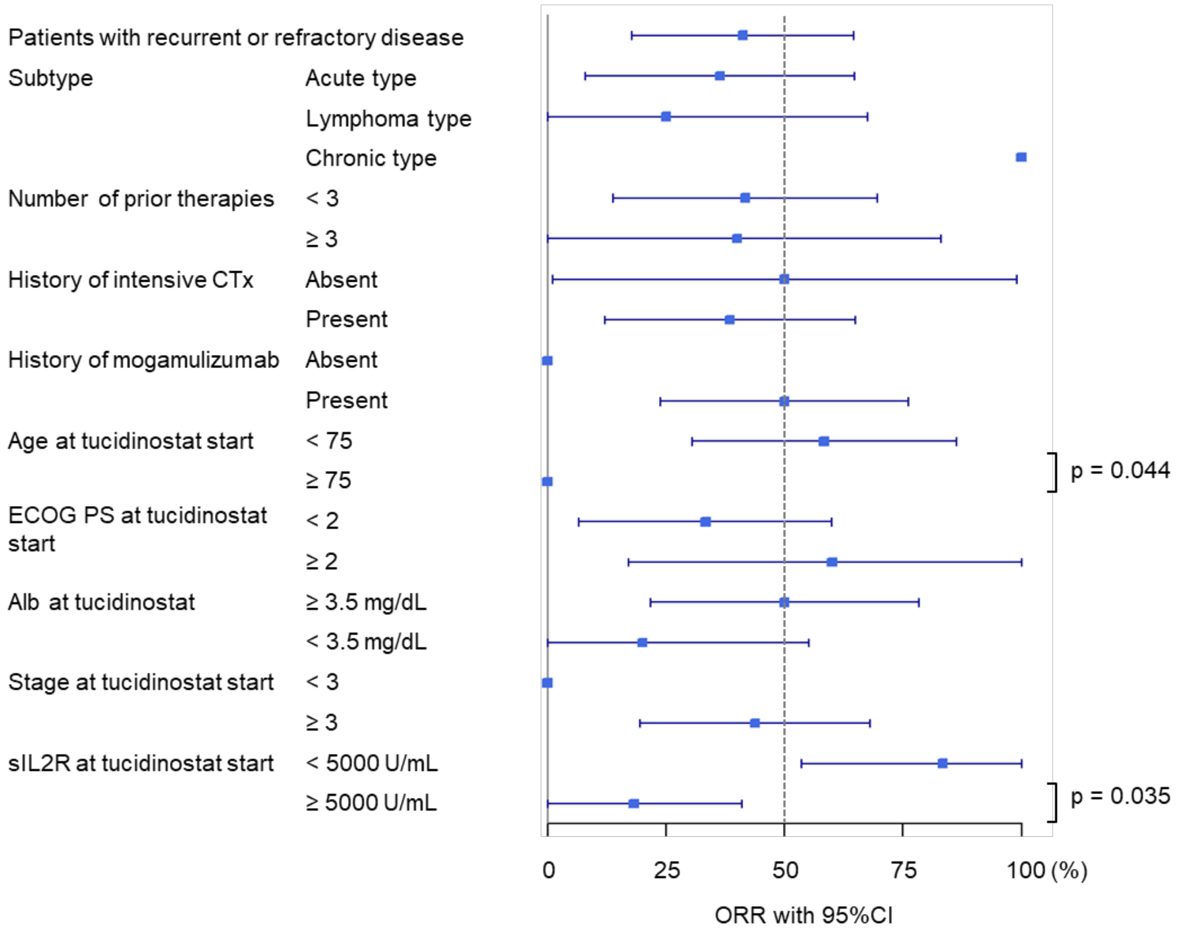

Figure S3. Objective response rate (ORR) by clinical variables following tucidinostat therapy for patients with recurrent/refractory ATL.

Forest plot depicting the ORR of tucidinostat therapy using clinical variables. The blue squares represent the estimated ORR, and the horizontal lines denote the 95% confidence intervals.

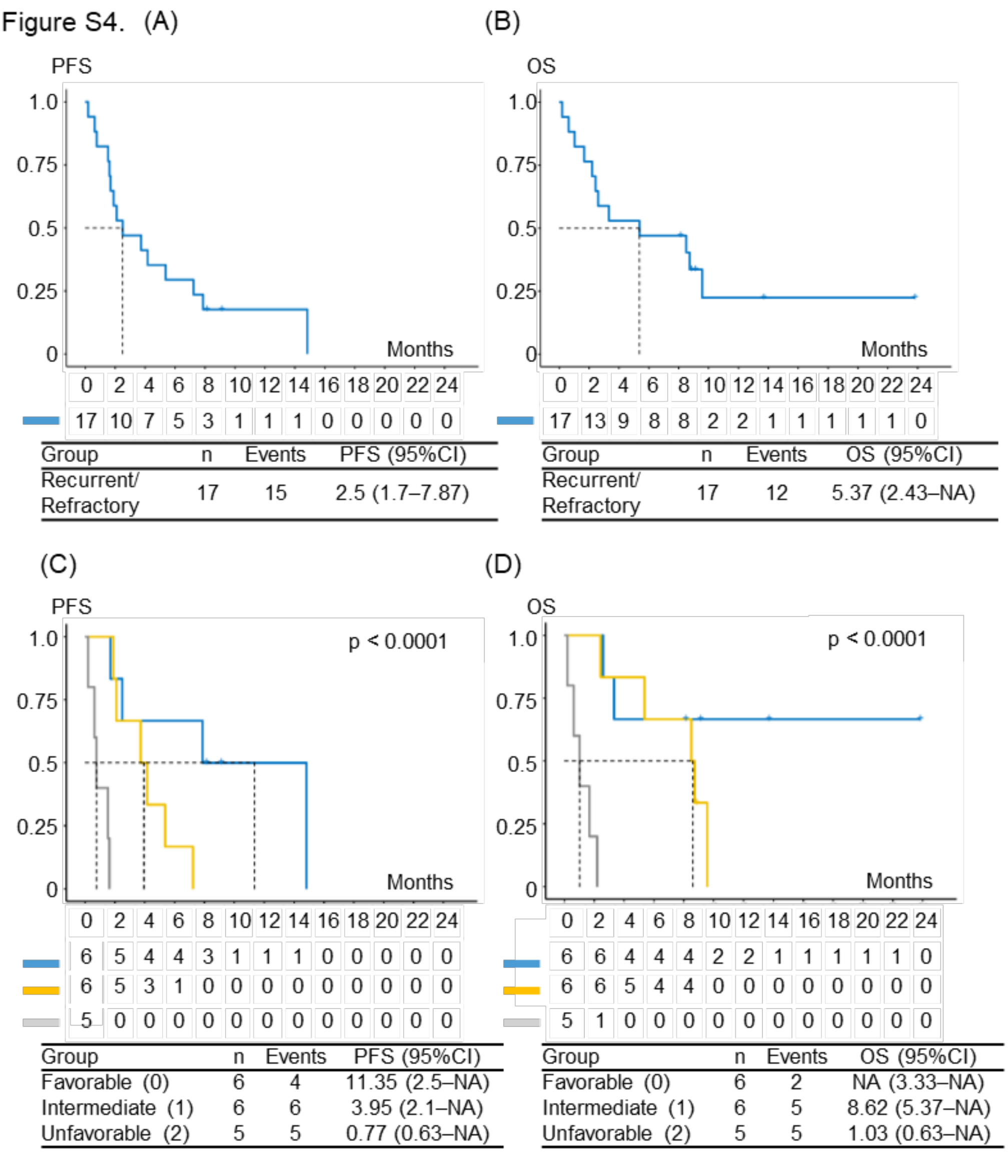

Figure S4. Efficacy of tucidinostat therapy on survival for patients with recurrent/refractory ATL.

(A) PFS for patients with recurrent/refractory ATL. (B) OS for patients with recurrent/refractory ATL. (C) PFS stratified by a prognostic model based on the total number of unfavorable factors (older age (≥75 years) and higher sIL-2R level (≥ 5000

U/mL)). Patients were categorized into three groups: favorable (0 factors), intermediate (1 factor), and unfavorable (2 factors). (D) OS stratified using the same prognostic model.
